# Fat People

**Published:** 1870-09

**Authors:** 


### FAT PEOPLE.

Not long ago, a gentleman of threescore, who had scarcely ever been sick in his life, thought he was too fleshy, and began to Bantamize. He succeeded famously, and boasted to his friends that he had got rid of ten pounds in a few weeks. A little later he was attacked with a painful and dangerous malady, from which he has been suffering more than a year.

If a man can sleep soundly, has a good appetite, with no unpleasant reminders after meals, the bodily habits being regular every day, he had better let himself alone whether he is as big as a hogshead or as thin and dry as a fence-rail.

Several cases of Bright's disease have been reported by medical men of reputation as a direct result of practicing Bantam's plan for getting lean. The very best and safest way to get rid of fat is to work it off; this may be aided by eating food which contains a large amount of nitrogen and a small amount of carbon. Nitrogenous food is that which gives strength, power to work, as lean meats; carbonaceous foods are those which make fat, such as cheese, potatoes, rice, corn, peas, beans, tapioca, arrow-root, corn starch, milk, sugar, syrup, and all oily and fat food. Raw fruits and berries largely eaten are great aids to reducing weight. But after all, the great reliance should be on exercise and work in the open air. Barclay, the great English pedestrian, who performed greater feats than Weston, lost ten pounds in two or three days' walking, and was never the worse for it.

